# Suppression of Molecular Inflammatory Pathways by Toll-Like Receptor 7, 8, and 9 Antagonists in a Model of IL-23-Induced Skin Inflammation

**DOI:** 10.1371/journal.pone.0084634

**Published:** 2013-12-27

**Authors:** Mayte Suárez-Fariñas, Robert Arbeit, Weiwen Jiang, Francesca S. Ortenzio, Tim Sullivan, James G. Krueger

**Affiliations:** 1 Laboratory for Investigative Dermatology, The Rockefeller University, New York, New York, United States of America; 2 Center for Clinical and Translational Science, The Rockefeller University, New York, New York, United States of America; 3 Idera Pharmaceuticals, Inc., Cambridge, Massachusetts, United States of America; Northwestern University Feinberg School of Medicine, United States of America

## Abstract

Psoriasis is a complex inflammatory disease resulting from the activation of T helper (Th) 1 and Th17 cells. Recent evidence suggests that abnormal activation of Toll-like receptors (TLRs) 7, 8 and 9 contributes to the initiation and maintenance of psoriasis. We have evaluated the effects of TLR antagonists on the gene expression profile in an IL-23-induced skin inflammation model in mice. Psoriasis-like skin lesions were induced in C57BL/6 mice by intradermal injection of IL-23 in the dorsum. Two TLR antagonists were compared: IMO-3100, an antagonist of TLRs 7 and 9, and IMO-8400, an antagonist of TLRs 7, 8 and 9, both of which previously have been shown to reduce epidermal hyperplasia in this model. Skin gene expression profiles of IL-23-induced inflammation were compared with or without TLR antagonist treatment. IL-23 injection resulted in alteration of 5100 gene probes (fold change ≥ 2, FDR < 0.05) including IL-17 pathways that are up-regulated in psoriasis vulgaris. Targeting TLRs 7, 8 and 9 with IMO-8400 resulted in modulation of more than 2300 mRNAs while targeting TLRs 7 and 9 with IMO-3100 resulted in modulation of more than 1900 mRNAs. Both agents strongly decreased IL-17A expression (>12-fold reduction), normalized IL-17 induced genes such as beta-defensin and CXCL1, and normalized aberrant expression of keratin 16 (indicating epidermal hyperplasia). These results suggest that IL-23-driven inflammation in mouse skin may be dependent on signaling mediated by TLRs 7, 8, and 9 and that these receptors represent novel therapeutic targets in psoriasis vulgaris and other diseases with similar pathophysiology.

## Introduction

Psoriasis is a chronic inflammatory disease of the skin, characterized by keratinocyte hyperplasia, dermal leukocyte infiltration and dermal vascular enhancement [1]. It affects approximately 2% of the population and almost 90% of individuals suffer from the most common form known as plaque psoriasis [2]. Immune cell infiltrates within psoriatic lesions predominantly consist of CD3+ Th1, Th17 cells and CD11c+ dendritic cells (DCs) [3], [4], [5]. The cytokines produced by these cells, such as tumor necrosis factor-α (TNFα), interferon-γ (IFNγ), IL-17, IL-22, IL-23, IL-12 and IL-1β, create an inflammatory cascade, contributing to the pathogenesis of psoriasis. This cytokine milieu further activates keratinocytes and other resident cutaneous cells and induces abnormal expression of antimicrobial peptides and other defensin genes [6].

The critical role played by the IL23/Th17 axis in psoriasis has been highlighted in recent studies [7],[8]. IL-23 is produced by antigen presenting cells such as DCs, and in addition to driving differentiation of naïve CD4+ T cell precursors towards the Th17 phenotype [9], IL-23 also stimulates survival and expansion of Th17 populations [10]. In turn, IL-17 produced by Th17 cells exerts direct regulatory control over the expression of defensins, S100 family proteins, and LL-37 [11],[12], all of which contribute to innate immune responses within skin. Lesional (LS) skin from humans exhibits higher expression of IL-23 in keratinocytes and dermal tissue in comparison to non-lesional

 (NL) and normal skin [13],[14]. The high efficacy of antibodies that target IL-23 and IL-17 further substantiates the integral role these cytokines play in psoriasis [15]. Studies performed in mice reveal IL-23-mediated inflammation to be highly dependent upon production of IL-17 [16]. Cutaneous IL-23 injections in mice result in epidermal hyperplasia and parakeratosis, somewhat reminiscent of the human psoriasis phenotype [17]. These observed changes make the IL-23 treated mouse a useful model for human skin inflammation. Although morphological similarities are readily visible, the extent to which there is genomic overlap between human psoriasis and the IL-23 treated mouse model remains to be elucidated.

Other mouse models with phenotypes that appear somewhat analogous to human psoriasis have been analyzed on a genomic level. A recent study performed novel transcriptomics-based comparisons between human psoriasis and five different psoriasiform mouse models [18]. Four transgenic models, K14-AREG, K5-STAT3C, K5-TGFβ1 and K5-Tie2, were investigated in addition to an imiquimod (IMQ)-induced model. The K14-AREG and K5-STAT3C both manifested inflammatory phenotypes via disruption of keratinocyte homeostasis, in turn causing increased cytokine release and a profound inflammatory response. Overexpression of human growth factor amphiregulin and a constitutive activation of a signaling component, Stat3, are the inciting events responsible for the K14-AREG and K5-STAT3C, respectively [19], [20]. The K5-Tie2 model, a result of a tyrosine kinase overexpression within basal keratinocytes, and the K5-TGFβ1 model, caused by overexpression of a latent form of transforming growth factor beta 1, both initiate inflammation via keratinocyte dysregulation, in conjunction with other mechanisms such as perturbance of the basement membrane and angiogenesis [21], [22]. IMQ, an agonist of TLRs 7 and 8, causes T cell infiltration and keratinocyte and vascular hyperplasia upon topical application [23]. For comparison, a human psoriasis transcriptome was extrapolated from differences in gene expression between psoriatic LS and normal skin using whole-genome microarray analysis. Transcriptomes for each mouse model were acquired in similar fashion, using naïve mouse skin as a control to compare against each inflammatory phenotype. Global correspondence of gene expression between human psoriasis and all five mouse models was deemed significant [18]. Further determination of ‘genomic fidelity’ between mouse inflammatory models and human psoriasis will aid in the identification of representative models of clinical psoriasis. Reliable animal models for the human psoriasis phenotype are valuable as the genetic and immunological underpinnings of the disease have not yet been fully delineated and the search for novel and improved treatments continues.

Potential therapeutic value has recently been demonstrated by a study showing that antagonism of TLRs 7, 8 and 9 reduced IL-23-induced epidermal hyperplasia and IL-17 expression [24]. Additionally, data collected from a Phase 2 clinical trial has shown a TLR 7 and 9 antagonist reduced PASI scores in psoriasis patients [25]. TLRs are transmembrane receptors that recognize pathogen-associated molecular patterns (PAMPs) and mediate innate immune defense against pathogens. TLRs 3, 7, 8 and 9 are all located in the endosome, however, TLR3, TLR7 and TLR8 bind RNA while TLR9 binds DNA containing unmethylated CG dinucleotides [26]. The expression and activity of TLRs 7, 8 and 9 is regulated by interactions between these receptors. Although TLR8 has been deemed nonfunctional in mice, recent evidence suggests it exerts regulatory control over other TLRs [27]. TLRs are mainly expressed on immune cells such as antigen presenting cells, with TLR7 and 9 on plasmacytoid DCs (pDCs) and B cells, and TLR8 on myeloid DCs (mDCs). A positive feedback loop of inflammation is created when these pDCs and mDCs are activated by immune complexes consisting of self-nucleic acids and LL-37 (cathelicidin), an antimicrobial peptide overexpressed in psoriatic lesions [28], [29]. Interactions between TLRs on DCs and immune complexes induces production of type I IFN and facilitates T cell autoreactivity, ultimately contributing to lesional tissue changes [30]. It appears that TLR7- and TLR9-signaling stimulates IL-23 secretion by DCs [31], [32], consequently up-regulating IL-17 production [33]. A TLR7/8 agonist used to treat skin abnormalities such as cancerous lesions, IMQ, has been shown to exacerbate psoriasis in patients [34]. A significant role for TLRs in psoriasis pathogenesis [35] is further supported by the finding that IL-23/IL-17 dependent features of clinical psoriasis were induced by topical application of IMQ [23]. 

In this study, we used a genome wide expression profile analysis to characterize the IL-23-induced model of skin inflammation. Comparison of global gene expression patterns as well as individual pathway analysis allowed us to determine how closely the IL-23-induced mouse model resembled the human psoriasis phenotype. For completeness, five previously analyzed mouse models [18] were included in comparison with the IL-23-induced mouse model and human psoriasis. Furthermore, treatment responses to two different TLR antagonists were evaluated in the IL-23-induced mouse model.

## Results

### Significant up-regulation of inflammatory cytokines in IL-23-induced inflammatory mouse model

Genetic changes associated with the IL-23-induced mouse phenotype were elucidated by comparison of full-thickness IL-23 injected skin with naïve mouse skin using mouse4302 Affymetrix gene array. As expected, IL-17A mRNA was up-regulated by 13 fold in the diseased model, in addition to other IL-17-regulated genes in keratinocytes such as CXCL1 by 120-fold, lipocalin (LCN2) by 53-fold, S100A8 by 36-fold and both defensin (Defb4) and S100A9 by 30-fold. Innate cytokines were also highly induced in the inflammatory model including IL-6, which was up-regulated by 95-fold, and members of the IL-1 family, namely, IL1-F5, F6, F8, F9 and IL1β, which increased by 55-fold. Keratin 16 mRNA increased by approximately 12-fold, which may directly correlate with the observed epidermal hyperplasia in the IL-23 mouse model. Surprisingly, interferon-γ mRNA exhibited an increase of almost 20-fold in addition to downstream interferon-response genes: CXCL11 by 29-fold; CXCL9 by 21-fold; and STAT1 by 4.7-fold. Using predefined cut-off values of fold-change (FCH)>2 and false-discovery rate (FDR)<0.05, we identified a total of 2346 up and 2762 down-regulated probe-sets, which encoded 1726 and 1775 unique genes, respectively. This profile of differentially regulated genes, which we will herein refer to as the IL-23-induced mouse transcriptome, can be found in Table S1.

### Significant correspondence between IL-23-induced mouse model of inflammation and human psoriasis

**Figure 1:**
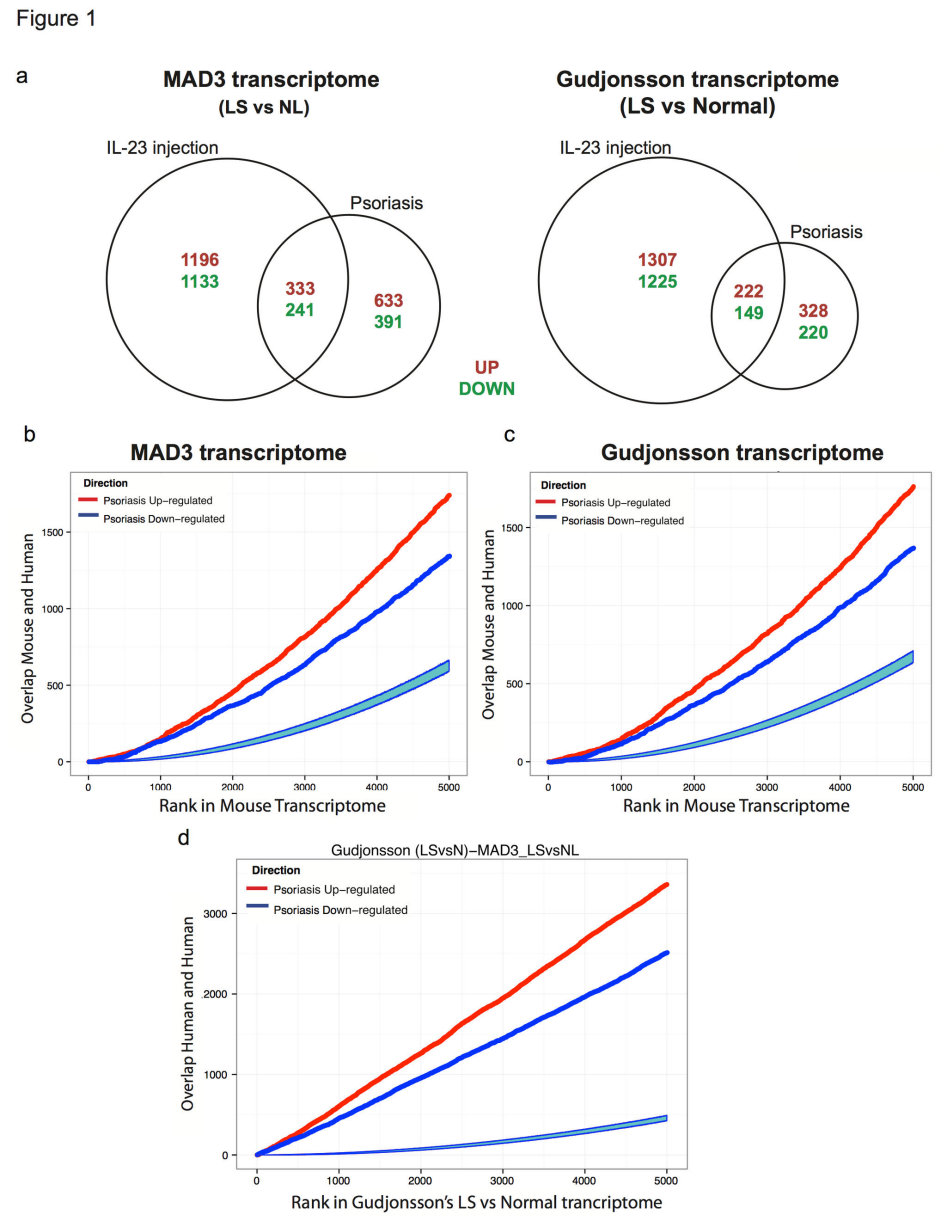
Murine IL-23 induced model of inflammation corresponds to the human psoriasis phenotype: Statistically significant overlap of DEGs and ranked gene lists. A. Venn diagrams illustrate relative overlap of orthologous DEGs between human psoriasis and the IL-23-induced mouse model. There are 10% more common DEGs between the IL-23 mouse model and MAD3 compared to that with Gudjonsson(LSvsNormal). B, C. Overlap between top k up- (red lines) and k down-regulated (dark blues lines) genes in the IL-23 transcriptome and the murine orthologs of the human MAD3 and Gudjonsson(LSvsNormal) transcriptomes, respectively, was estimated for k=1,…5000.. D. As a reference, the overlap between both human transcriptomes was analyzed in similar fashion. Statistically significant overlap is seen for all three depictions of ranked gene overlap, as the light blue regions represent degree of overlap expected under the null hypothesis of random overlap.

In order to compare how well the IL-23-induced model represented human psoriasis, analysis was performed using two previously published human psoriasis transcriptomes. One of the transcriptomes, MAD3, is meta-analysis-derived and based on differences in gene expression between LS and NL skin across three independent profiling studies in humans [36]. The other human transcriptome, which will herein be referred to as Gudjonsson(LSvsNormal), is based on differences in gene expression between psoriasis lesions and normal healthy control skin [37], and served as the representative human psoriasis transcriptome in a previous comparative analyses with five mouse models [18]. Analysis of concordance between human and the IL-23-induced mouse model revealed 25% of differentially expressed genes (DEGs) in the IL-23 transcriptome were present in MAD-3, compared to 15% in the Gudjonsson(LSvsNormal) (Figure 1A). As choice of cut-offs can influence the intersections between studies’ DEGs [38], we used previously described methods [18] to compare human and mouse transcriptomes using ranked gene lists. The 5000 most strongly up-regulated and 5000 most strongly down-regulated genes were identified in the IL-23-induced mouse transcriptome, and then ranked by estimated FCH between LS and normal. For each rank k, the top k murine genes were identified and the overlap between them and the murine orthologs of the human transcriptomes was determined. There was statistically significant overlap of top k up- and down-regulated transcripts for the IL-23 mouse and both human transcriptomes (Figure 1B, 1C). Comparison between the human transcriptomes themselves showed high degree of overlap, with psoriasis-increased ranked gene lists exhibiting greater overlap than psoriasis-decreased (Figure 1D). Global similarity between the IL-23-induced mouse model and human psoriasis was further demonstrated with gene-set enrichment analysis (GSEA). Using a previously described method [38], ten previously published human psoriasis transcriptomes were treated like individual gene sets in order to quantify the extent to which up- and down-regulated genes correlated with ordered DEGs from the IL-23-induced mouse (Table 1). Excluding the methylation in psoriasis transcriptome, normalized enrichment scores (NES) spanned 2.04 - 2.41 for up and -2.77 to -1.88 for down-regulated DEGs in the IL-23-induced LS skin, indicating significant enrichment of human psoriasis gene sets in the IL-23-induced phenotype.

**Table 1:** GSEA analysis for the IL23-induced phenotype in mice versus psoriasis transcriptomes in human.

Psoriasis Transcriptomes	Up			Down				Reference
	Size	ES**^*1*^**	NES**^*2*^**	Size	ES	NES	CS**^*3*^**	
Gudjonsson ’09 (LSvsNL)	425	0.71	2.41	214	-0.58	-2.43	0.65	[52]
Gudjonsson ’10 (LSvsNormal)	482	0.70	2.43	336	-0.59	-2.54	0.64	[37]
MAD5	579	0.68	2.37	390	-0.58	-2.57	0.63	[36]
MAD3	899	0.66	2.33	608	-0.59	-2.70	0.63	[36]
Yao’08	820	0.68	2.40	730	-0.55	-2.59	0.62	[40]
Suárez-Fariñas ’10	500	0.67	2.34	633	-0.52	-2.43	0.60	[38]
NGS (Jabbari/SF’12)	895	0.62	2.15	748	-0.58	-2.77	0.60	[53]
Suárez-Fariñas +	1362	0.58	2.04	949	-0.53	-2.54	0.55	[39]
Zhou ‘03	220	0.68	2.27	344	-0.42	-1.88	0.55	[54]
Methylation in Psoriasis (LSvsNormal)	379	-0.65	-2.22	457	0.34	1.55	-0.49	[55]

Size indicates the number of genes in each transcriptome

^1^ ES = enrichment score

^2^ NES = normalized enrichment score

^3^ CS = connectivity core, calculated as **½**(ES(Up)-ES(Down))

### Comparative analysis of 6 inflammatory mouse models reveals differential expression of inflammatory pathways

**Figure 2:**
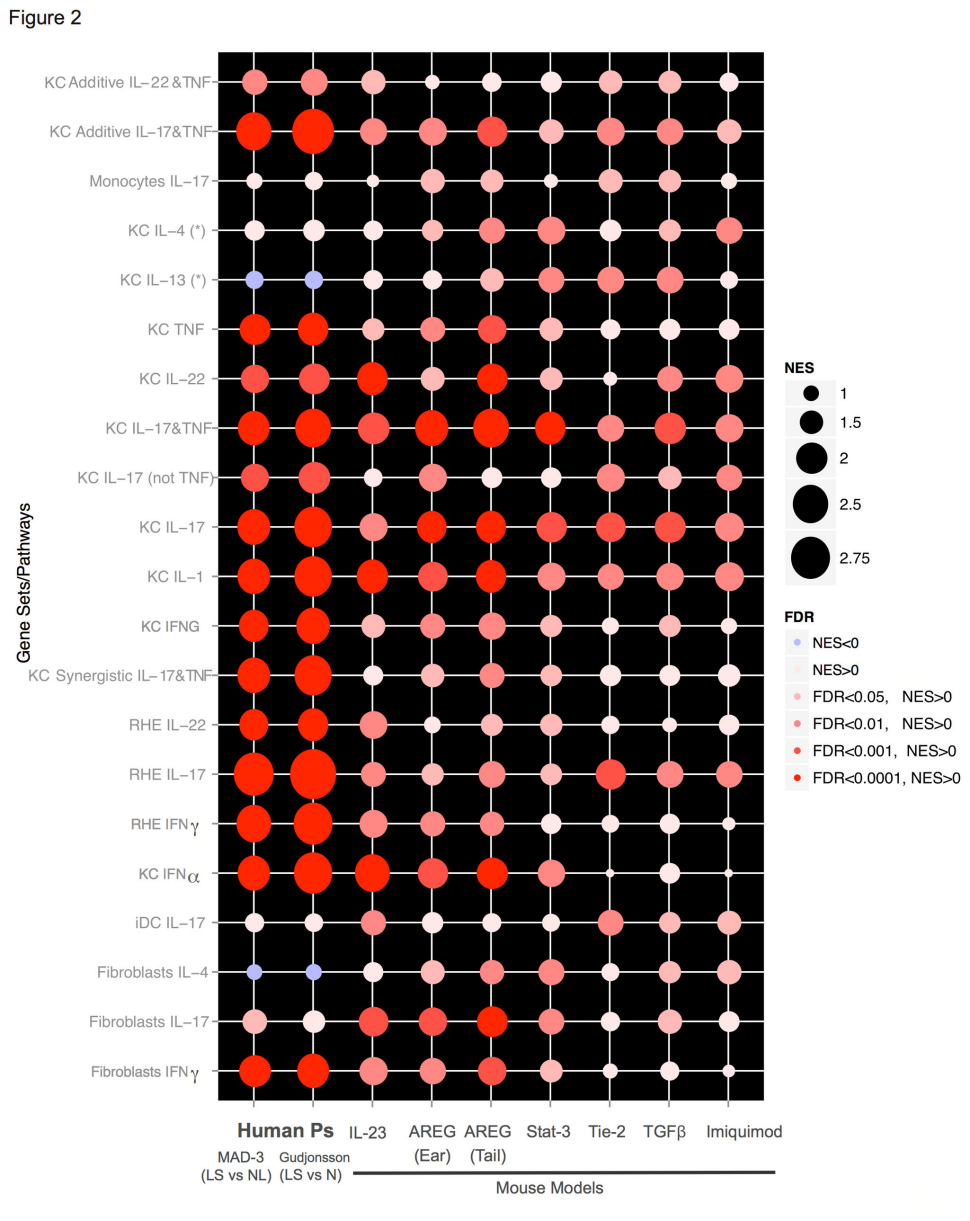
Pathways enriched in human psoriasis and inflammatory mouse model transcriptomes with GSEA. Degree of enrichment of key inflammatory pathways implicated in psoriasis pathogenesis compared in human and murine transcriptomes is portrayed by the bubbles representing normalized enrichment score (NES) and false discovery rate (FDR) values. Six inflammatory mouse model transcriptomes; IL-23-induced (IL-23), K14-AREG (AREG for both ear and tail), K5-Stat3c (Stat3), K5Tie2 (Tie-2), K5-TGFβ1 (TGFβ) and IMQ as well as two human psoriasis transcriptomes; MAD3 and Gudjonsson(LSvsNormal), were queried with sets of cytokine-treated keratinocyte, monocyte, fibroblast, inflammatory DC and reconstituted human epidermis (RHE) pathways.

We applied the previously described method of gene rank overlap analysis to five additional mouse models to evaluate their correspondence with the human psoriasis phenotype as represented by MAD3. In agreement with previously described results [18], we found the K5-Tie2, IMQ, K14-AREG, K5-Stat3C and K5-TGFbeta1 mouse models all shared expression patterns with human psoriasis. Although the K14-AREG mouse was the closest of the transgenic models to the human phenotype, side by side comparison revealed the IL-23 mouse resembled the expression patterns in human psoriasis with greatest fidelity overall (Figure S1). Various core inflammatory pathways that are represented in the overall gene set were analyzed using GSEA (Figure 2). The Gudjonsson(LSvsNormal) showed slightly exaggerated expression of IL-17 driven pathways in comparison to the MAD3, which had overall greater correlation with the expression profiles of all mouse models. The IL-23 mouse model best matched the attenuated Th2 profile seen in human psoriasis, whereas the amphiregulin model showed a slightly more robust expression of Th2 pathways. Interestingly, the IL-17 and TNF axes were well-represented in the IL-23 and amphiregulin models, however, the IL-23 model displayed stronger representation of IFNα induced genes, in better accord with human phenotype. Additionally, genes regulated by IL-22 in keratinocytes were also more correctly represented within the IL-23 model. Overall, the IL-23 model best reflected the cytokine-mediated processes found in human psoriasis, although it clearly does not encompass the full psoriasis genotype.

### Treatment with TLR antagonists regulates IL-23-induced gene expression

**Figure 3:**
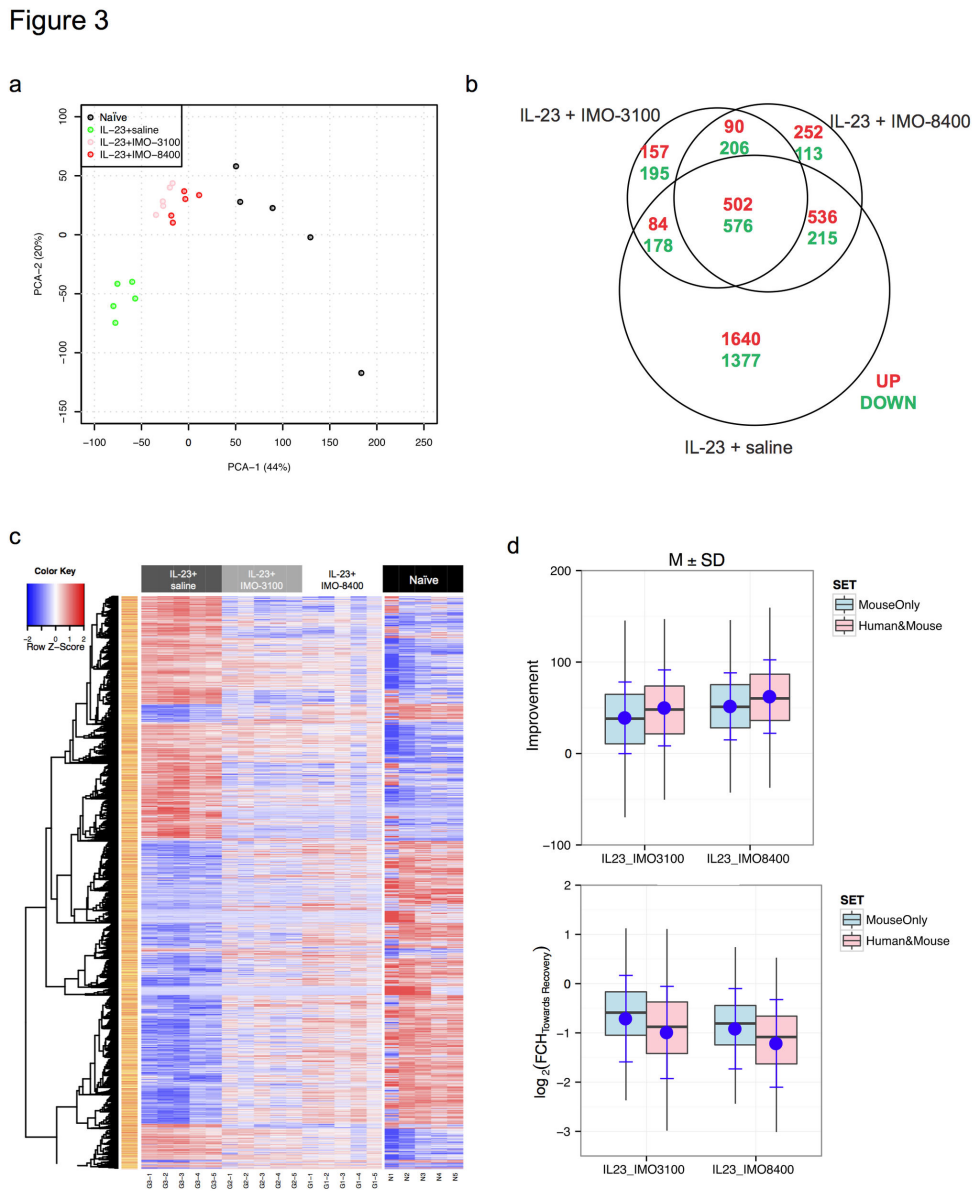
IL-23-induced gene expression profile shifts towards recovery with TLR antagonism. A. Differences in gene expression in naïve, IL-23, IL-23+IMO-3100 and IL-23+IMO-8400 treated mice were analyzed using principal component analysis. Partial normalization of gene expression patterns in the IL-23-induced mouse model was seen following treatment with TLR antagonists, with IMO-8400 exerting a slightly greater effect. B. IMO-8400 modulated 10% more of the IL-23 altered genes compared to IMO-3100, indicating additive effects of TLR8 antagonism in addition to TLR7 and 9. C. Shifts in gene expression profiles for IL-23 treated mice towards naïve mice following treatment with TLR antagonists are displayed in heatmap. Of the genes that shifted towards recovery with TLR antagonism, a greater proportion consisted of shared human and mouse genes rather than those that were unique murine genes.

Immunological pathway modifications by two different TLR antagonists were measured in the IL-23-induced mouse model, which was chosen for its histological [24] and molecular resemblance to human psoriasis. Skin lesions were induced in mice by intradermal injection of IL-23 into the dorsum, later followed by subcutaneous injections of either IMO-3100 (TLR7/9 antagonist) or IMO-8400 (TLR7/8/9 antagonist), distal to the IL-23 injection site. Principal component analysis based on the acquired microarray data illustrates that both TLR antagonists exert significant effects on gene expression patterns exhibited by the IL-23-induced mouse model. Expression patterns exhibited by the IMO-3100 and the IMO-8400 treated mice exhibited a shift towards the expression profile of naïve mice, with slightly more profound effect seen in the IMO-8400 treated cohort (Figure 3A). Analysis of gene overlap was accomplished by comparison of the naïve mouse gene expression profile with that of the IL-23-induced phenotype both pre- and post-treatment with both TLR antagonists. Using a cut-off level of FDR<0.05 and FCH>2, it was found that IMO-3100 modulated 26% of the IL-23-regulated genes, while the additive effect of TLR8 antagonism in IMO-8400 was associated with 36% alteration of IL-23 genes (Figure 3B). Ingenuity pathway analysis revealed that the additional antagonism of TLR8 with IMO-8400 was linked to several canonical pathways including immune cell trafficking, inflammatory response and antimicrobial response pathways. Upstream regulators were largely inflammatory in nature, including gene networks involved in both innate and adaptive immune responses as well as IFNγ signaling and components of the IL-17 pathway such as IL-21. A nearly inverse pattern of gene expression for IL-23-injected mice and naïve mice is portrayed by the heatmap in Figure 3C. The disparity in gene expression between disease and naïve state is visibly reconciled to some extent by treatment with TLR antagonists, evidenced by post-treatment expression profiles bearing greater resemblance to the naïve rather than the inflammatory state (Figure 3C). TLR antagonist treatment resulted in an average FCH of 1.94 towards recovery for genes altered as a result of IL-23-induced inflammation, with a 53.21% improvement seen with administration of IMO-8400. Overall, 52% and 39% of genes in the IL-23-induced phenotype decreased by >50% in the IMO-8400 and IMO-3100 groups, respectively. Interestingly, genes shared between the IL-23-mouse and the MAD3 human psoriasis transcriptomes experienced 10 points higher improvement compared with genes unique to the mouse model (62.26% vs. 51.6%) as seen in Figure 3D, upper panel. A FCH towards recovery of 2.32 was observed for human and mouse orthologous genes vs. 1.89 for purely mouse genes (Figure 3D, lower panel). Collectively, these results indicate TLR7, 8, and 9 antagonism partially resolves IL-23-induced inflammation.

### Suppression of inflammatory pathways by TLR antagonism

Fold changes in inflammatory genes highly implicated in psoriasis pathogenesis determined by microarray are displayed for the IL-23-induced mouse model as well as treatment groups in Table 2. Treatment with each TLR antagonist significantly suppressed many of the inflammatory genes, with substantial reduction in IL-17A mRNA (>12-fold) as well as β-defensin, CXCL1, CXCL2, CXCL3, LCN2, and other IL-17 pathway molecules, including the IL-21 receptor and IL-12Rβ1. Additionally, IL-6, an up-stream regulator of Th17 development, was reduced by 98-fold along with IFNγ, which decreased by 8-fold and 11.5-fold with IMO-3100 and IMO-8400, respectively. IFNγ pathway genes like CXCL9 and IL-12Rβ1 were also reduced. IL1 decreased 6-fold with IMO-3100 and 12-fold with IMO-8400 and decline in NFkB mRNA was observed. Several cytokine receptors that signal though CD132 and JAK3 were down-regulated. The results of GSEA analysis, which evaluated global treatment effects for the entire set of genes, yielded similar results. Down-regulation of genes overexpressed in keratinocytes cultured with IL-17, IFNγ and IL1 as well as the JAK-Stat pathway and IL-23, IL-12 and IL-27 canonical pathways following IMO-8400 treatment (Table 3). Interestingly, the type I diabetes pathway was found to be down-regulated following treatment as well.

**Table 2:** Selected list of murine genes orthologous to inflammatory genes implicated in psoriasis pathogenesis that are regulated by TLR antagonists.

Symbol	Description	Fold ChangeIL-23 +Saline	Fold ChangeIL-23 +IMO-3100	Fold ChangeIL-23 +IMO-8400
**Defb3**	defensin beta 3	1012.96	-20.76	-16.62
**Cxcl1**	chemokine (C-X-C motif) ligand 1	120.8	-18.62	-7.54
**Cxcl1**	chemokine (C-X-C motif) ligand 1	109.08	-15.37	-6.50
**Il6**	interleukin 6	94.80	-98.77	-98.23
**Il1b**	interleukin 1 beta	55.03	-5.94	-12.11
**Lcn2**	lipocalin 2	53.35	-5.83	-3.87
**Cxcl1**	chemokine (C-X-C motif) ligand 1	47.74	-13.51	-7.05
**S100a8**	S100 calcium binding protein A8 (calgranulin A)	36.88	-3.33	-2.11
**Defb4**	defensin beta 4	30.65	-20.58	-29.92
**S100a9**	S100 calcium binding protein A9 (calgranulin B)	30.44	-2.18^1^	-1.50^2^
**Cxcl11**	chemokine (C-X-C motif) ligand 11	29.81	-29.61	-28.68
**Il24**	interleukin 24	27.08	-27.08	-27.08
**Il1f6**	interleukin 1 family, member 6	23.85	-1.10^2^	-1.12^2^
**Cxcl9**	chemokine (C-X-C motif) ligand 9	21.68	-16.00	-20.66
**Ifng**	interferon gamma	19.77	-8.45	-11.57
**Tbx21**	T-box 21 (T-bet)	13.66	-2.45	-1.76^1^
**Cxcl10**	chemokine (C-X-C motif) ligand 10	13.08	-2.50	-2.98
**Il17a**	interleukin 17A	12.89	-12.84	-12.55
**Krt16**	keratin 16	11.92	-3.23	-2.07
**Tnfsf10**	tumor necrosis factor (ligand) superfamily, member 10	8.69	-5.44	-7.95
**Cxcl2**	chemokine (C-X-C motif) ligand 2	8.05	-5.10	-7.44
**Ccr2**	chemokine (C-C motif) receptor 2	7.26	-13.83	-13.84
**Il1f8**	interleukin 1 family, member 8	6.97	1.04^2^	-1.04^2^
**Ccr2**	chemokine (C-C motif) receptor 2	6.41	-4.97	-6.35
**Stat1**	signal transducer and activator of transcription 1	4.69	-1.87^2^	-3.60
**Il1f9**	interleukin 1 family, member 9	3.81	-1.36^2^	-1.21^2^
**Il1f5**	interleukin 1 family, member 5 (delta)	3.53	-1.06^2^	1.02^2^
**Il21r**	interleukin 21 receptor	3.06	-2.47	-2.85
**Cxcr3**	chemokine (C-X-C motif) receptor 3	3.04	-1.80^2^	-1.77^2^
**Il1f5**	interleukin 1 family, member 5 (delta)	2.46	1.01^2^	1.08^2^

^1^ FDR is not statistically significant

^2^ FCH does not reach significance threshold

**Table 3:** GSEA analysis for the effect of IMO-8400 over canonical pathways (C2 collection) and psoriasis related pathways (a selection of the significant pathways is presented).

**MolSigDb C2 collection**	N	ES	NES	FDR
IL-23 Pathway (PID)	31	-0.84	-2.57	<10^-4^
Cytokine Receptor Interaction (KEGG)	191	-0.61	-2.54	<10^-4^
Cell Cycle (Reactome)	344	-0.55	-2.46	<10^-4^
IL-12 Pathway (PID)	56	-0.68	-2.35	<10^-4^
Graft Versus Host Disease (KEGG)	23	-0.80	-2.24	4.94E-05
IL-27 Pathway (PID)	23	-0.77	-2.23	4.09E-05
Type I Diabetes Mellitus (KEGG)	24	-0.75	-2.17	1.01E-04
Natural Killer Cell Mediated Cytotoxicity (KEGG)	93	-0.58	-2.15	3.23E-04
Allograft Rejection (KEGG)	22	-0.76	-2.12	5.30E-04
Chemokine Signaling Pathway (KEGG)	161	-0.50	-2.04	0.0017
JAK-STAT Signaling Pathway (KEGG)	113	-0.52	-2.02	0.0019
Interferon Signaling (Reactome)	117	-0.51	-2.00	0.0023
Toll Endogenous Pathway (PID)	23	-0.70	-1.99	0.0029
Toll-Like Receptor Signaling Pathway (KEGG)	81	-0.53	-1.96	0.0046
STAT3 Pathway (ST)	11	-0.81	-1.90	0.0076
IL-6 Pathway (PID)	44	-0.53	-1.76	0.0293
**Psoriasis-related gene sets**				
Genes down-regulated after 2 weeks of IL-17 antagonist Ixekinumab	675	-0.57	-2.70	<10^-4^
Up-regulated by IFN**γ**in Normal Skin (JID, 2012)	722	-0.53	-2.54	<10^-4^
KC IL-17 Up	41	-0.72	-2.36	<10^-4^
RHE IFNγ Up	178	-0.56	-2.33	<10^-4^
Fibroblasts IL-17 Up	37	-0.73	-2.29	<10^-4^
Additive IL-17 & IL-22 KC	19	-0.74	-2.02	3.51x10^-4^
Synergistic IL-17 & IL-22 KC	26	-0.67	-2.00	4.05 x10^-4^
KC IFNα Up	24	-0.69	-1.98	6.19 x10^-4^
Additive IL-17 & TNFα in KC	165	-0.43	-1.76	0.0062
Synergistic IL-17 & TNFα in KC	128	-0.38	-1.51	0.0386
KC TNF Up	460	-0.31	-1.42	0.0633
KC IFN**γ**Up	872	-0.28	-1.33	0.0957

N = number of genes detected in each pathway

## Discussion

While several different inflammatory models in mouse skin have shown some features that are consistent with human psoriasis, it is clear that not all features of disease are represented within the available models. At present, psoriasis is best defined by the array of genes that are dysregulated in diseased tissue, identified by comparison of LS to NL tissue across multiple studies and with a meta-analysis of different studies consistently showing greater than 1000 genes, collectively defining the psoriasis transcriptome [36]. The study with the largest number of samples, Suárez-Fariñas et al. [39], has detected more than 4000 genes that are dysregulated by criteria of greater than 2 FCH and a FDR <0.05. In this study, we first sought to determine the extent by which the IL-23 model reflects molecular and inflammatory pathways expressed in human psoriasis and secondly, to determine how this model relates to other inflammatory models where transcriptome profiles have been made available. Within this context, the key inflammatory pathways in psoriasis, such as IL-23 stimulated activation of IL-17 and downstream genes, are well represented. Also, the IL-23 model produces the least amount of Th2 activation, which in conjunction with Th17, Th22 and IFNγ are the defining elements of inflammation in psoriasis. High expression of IFNα related genes in LS skin has further implicated an important role for Type I IFNs in pathogenesis of psoriasis [40], [41].

TLR antagonism may represent a strategy for regulating the complex inflammatory environment in skin caused by psoriasis. A previously published study by Jiang et al., examined a TLR7, 8, and 9 antagonist in the IL-23 mouse model, focusing on psoriasis related cytokines as measured by RT-PCR [24]. The transcripts identified in that paper confirmed many of the cytokines that were detected in this study using gene array analysis and thus additional confirmation was rendered unnecessary. The data presented here extends the analysis initiated by Jiang et al., by measuring the entire IL-23 mouse transcriptome, including the regulation of many genes under cytokine-driven pathways. Use of gene arrays has permitted a deeper analysis of the inflammatory pathways that may be regulated by TLRs. An earlier study that indicated that TLR7 may be involved in psoriasis pathogenesis made the observation that application of IMQ could induce psoriatic lesions at sites of inflammation. The mechanism of this was suggested to be through activation of TLR7 on pDCs, leading to increased production of IFNs, with downstream effects on several inflammatory pathways regulated by IFN-induced genes [42]. pDCs also express TLR9, which although differs in which ligands it binds, shares a similar mode of endosomal transport and signaling pathway with TLR7 [43]. Hence, if activated, TLR9 might play a similar pathogenic role in psoriatic inflammation. In contrast, TLR8 is expressed mainly by mDCs [44], which are the dominant cell population in psoriasis lesions [45], and where activation of this TLR would be predicted to activate NFkB responsive pathways [46], which may include IL-23 production from DCs. Evidence that TLR7, 8 and 9 may participate in psoriasis pathogenesis is also suggested by the ability of LL-37-RNA and DNA complexes to activate pDCs and mDCs. Normally, the interaction between TLRs with endocytosed viral nucleic acids results in activation of mDCs and pDCs. In psoriasis, self-DNA and -RNA may be bound by LL-37, conferring protection against extracellular degradation and consequently allowing access to endosomal TLRs. Complexed DNA and LL-37 activates pDCs via TLR9, resulting in IFNα secretion [29]. Alternatively, self-RNA and cathelicidin complexes are also able to directly stimulate pDCs by binding to TLR7 and can also trigger mDCs through activation of TLR8 [28].

Another consideration is that of the role of TLRs in keratinocytes. In addition to producing elevated levels of cathelicidin, keratinocytes in psoriatic LS skin have been found to express significantly higher levels of TLR9 mRNA in comparison to NL psoriatic skin or that of atopic dermatitis. Additionally, when cultured with LL-37, keratinocytes further increased expression of TLR9 mRNA in vitro [47]. Activation of TLR3, 4, 5, and 9 in keratinocytes with various PAMPs has led to nuclear translocation of subunit p65 of NFkB in vitro [46]. The observed TLR activation in keratinocytes and subsequent triggering of NFkB, offers a potential mechanism by which keratinocytes may participate in IL-17 and TNF regulated inflammatory pathways. Keratinocytes respond to IL-17 with an up-regulation of neutrophil-attracting chemokines as well as CCL20, which interacts with CCR6+ cells including mDCs and Th17 cells that subsequently may become part of the lesional environ [48]. Although the exact role of TLRs in keratinocytes is not yet fully understood, further study is clearly warranted. Recently, the TLR7 and 9 antagonist used in this study, IMO-3100, was tested in a Phase 2 psoriasis treatment trial [25]. Although prior examination of a TLR7, 8, and 9 antagonist has been conducted in the IL-23 mouse model [24], no study prior to this has allowed for characterization of how gene circuits may be differentially affected by TLR7 and 9 vs. TLR7, 8, and 9 antagonism. Results presented herein suggest targeting TLR7, 8, and 9 impacts a broader array of IL-23-induced inflammation pathways than does targeting TLR7 and 9. This may also translate into future trials where targeting TLR7, 8, and 9 may be more efficacious in treating psoriasis. Additionally, although results of the clinical trial of the TLR7/9 antagonist are still being analyzed, transcriptomic data from the IL-23 model herein provides biomarker pathways that may be analyzed in psoriasis patients undergoing trials with TLR antagonists.

The shift in disease-associated gene expression of IL-17 and IL-23 towards normal levels in the IL-23 mouse following treatment with TLR antagonists suggests a potential role for TLRs in the psoriatic inflammatory cascade. It appears that TLR-regulated innate immune pathways may be an important facet of the cutaneous immune system in normal individuals [35]. Additionally, the involvement of various immune cell types in TLR signaling and the potential utility of TLR antagonists in cutaneous inflammatory diseases further necessitates that greater efforts be made in understanding the roles that these endosomic receptors fulfill within skin.

## Materials and Methods

### Animals

All protocols were approved by the Idera Institutional Animal Care and Use Committee. Female C57BL/6 mice, age of 6 weeks, were purchased from The Jackson Laboratory (Bar Harbor, ME). Mice were housed at the Idera Pharmaceuticals, Inc. animal facility for 1 week before initiating the study. All protocols were approved by the Idera Institutional Animal Care and Use Committee (n=5 per group).

### Induction of disease

Induction of lesions on dorsal skin was achieved by daily intradermal injection of recombinant murine IL-23 (3 µg, eBioscience, San Diego, CA) from day 1 to 4 in 100μl PBS. IL-23-treated mice were injected subcutaneously at a distal site with 15 mg/kg of each antagonist in 100μl PBS or, with 100μl PBS on day 4, 5 and 6 (n=5 per group). All mice were euthanized on day 7 and skin samples at the IL-23 injection site were collected for evaluation.

### Synthesis and purification of TLR antagonists

The antagonist oligonucleotides IMO-3100 and IMO-8400 were synthesized and purified as described earlier [49], [50] and contained < 0.075 EU/mg of endotoxin measured by the Limulus assay (Bio-Whittaker, Walkersville, MD). 

### Microarray Hybridization

Skin biopsies were stored in RNA Later at –20°C until used. Skin total RNA was isolated using RNeasy Mini kit (Qiagen, Valencia, CA) by a modified protocol. Briefly, 20mg of skin samples were homogenized in 700μl QIAzol Lysis reagent (Qiagen), followed by 140μl chloroform (Sigma, St. Louis, MO) and aqueous phase was collected after centrifugation at 12000 ×g for 15 minutes at 4°C. Absolute ethanol was added to the aqueous phase at 1.5 volume, mixed and loaded on to RNeasy Mini spin columns. Total RNA was purified according to the manufacture’s suggestion and later hybridized to GeneChip *mouse4302* (Affymetrix, Santa Clara, CA). Raw data have been deposited in NCBI’s Gene Expression Omnibus and are accessible through accession number GSE50400.

### Statistical analysis

Affymetrix (Santa Clara, CA) CEL files were scanned using software packages Harshlight [51] and arrayQualityMetrics from R/Bioconductor (www.bioconductor.org). Expression values (in log_2_-scale) were obtained using the GCRMA algorithm. Genes with expression higher than 2 in at least 3 samples and standard deviation of 0.1 were included in the statistical analysis. To identify DEGs moderated t-tests were used in the *limma* package framework. Resultant P-values were adjusted for multiple hypotheses using the Benjamini–Hochberg procedure, which controls the FDR. The cutoffs used to determine DEGs were FDR<0.05 and FCH>2. Annotation, including orthologs between human and mouse, were retrieved using R’s package biomart. 

The overlap between the top k genes in the murine transcriptome and their orthologs on the published human transcriptome was determined for k=1,…,5000. For each rank k, the top k murine genes were identified and the overlap between them and the mouse orthologs of the human transcriptomes was determined. Confidence Interval for the null hypothesis of random overlap between human and mouse was estimated via simulations. For each rank k (k=1,…K) , k genes in the murine transcriptome were randomly selected without replacement, and the overlap with the murine orthologs of the humans transcriptome was calculated. The empirical distribution of the overlap under randomness was estimated by repeating this procedure 5000 times.

## Supporting Information

Figure S1 **Overlap of ranked gene sets between mouse models and MAD3.** Ranked gene overlap analysis was performed for 5 previously published mouse models and the IL-23 mouse model, using MAD3 as the human psoriasis reference transcriptome. The red and dark blue lines in each figure respectively represent overlap between top and bottom ranked human orthologs from the murine model transcripts with MAD3. Light blue regions represent overlap as predicted under the null hypothesis. Results correlated with those previously described [18], except that the IL-23 model included in our analysis exhibited slightly superior overlap of up-regulated genes with human, compared to the K14-AREG and K5-TGFβ1 models, which otherwise overlap best with psoriasis vulgaris. With respect to down-regulated transcripts, the K14-AREG and K5-Tie-2 models are roughly comparable to the IL-23 model.
(TIF)

Table S1 **The IL-23-induced mouse model transcriptome.**
(XLSX)
