# Supplementary figures and images for: Suppression of Molecular Inflammatory Pathways by Toll-Like Receptor 7, 8, and 9 Antagonists in a Model of IL-23-Induced Skin Inflammation
Source: PLoS One. 2013 Dec 27;8(12):e84634. doi: 10.1371/journal.pone.0084634 (PMC3874038; doi:10.1371/journal.pone.0084634)

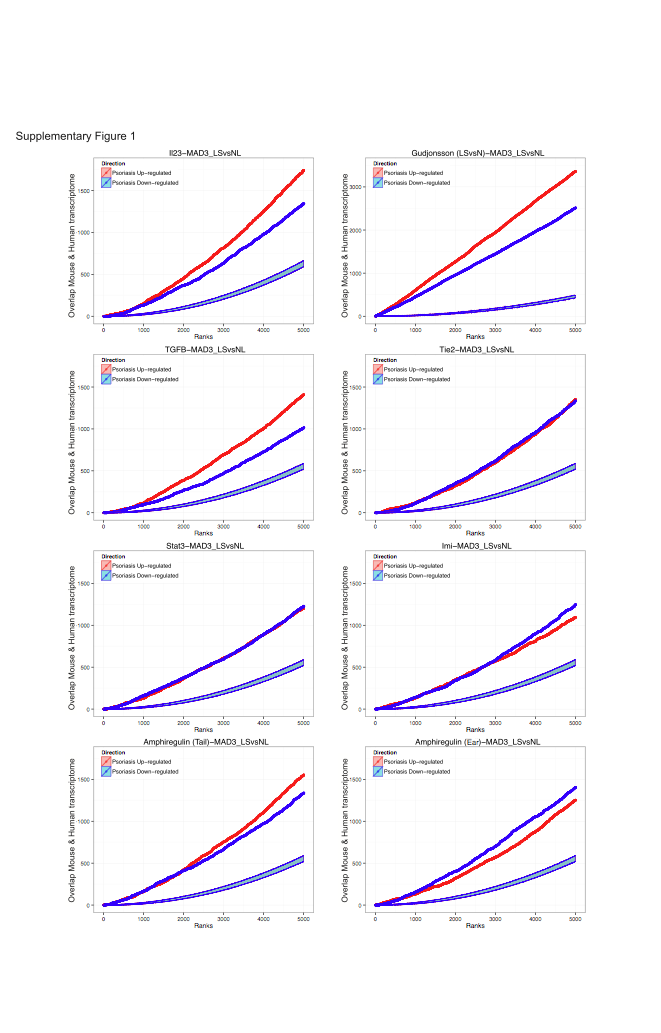

Supplement: Figure S1 — Overlap of ranked gene sets between mouse models and MAD3. Ranked gene overlap analysis was performed for 5 previously published mouse models and the IL-23 mouse model, using MAD3 as the human psoriasis reference transcriptome. The red and dark blue lines in each figure respectively represent overlap between top and bottom ranked human orthologs from the murine model transcripts with MAD3. Light blue regions represent overlap as predicted under the null hypothesis. Results correlated with those previously described [18], except that the IL-23 model included in our analysis exhibited slightly superior overlap of up-regulated genes with human, compared to the K14-AREG and K5-TGFβ1 models, which otherwise overlap best with psoriasis vulgaris. With respect to down-regulated transcripts, the K14-AREG and K5-Tie-2 models are roughly comparable to the IL-23 model. (TIF) [file pone.0084634.s001.tif]
